# From powerlessness to recognition the meaning of palliative care clinicians’ experience of suffering

**DOI:** 10.1080/17482631.2020.1852362

**Published:** 2020-11-29

**Authors:** Mélanie Vachon, Alexandra Guité-Verret

**Affiliations:** a Psychology Department, Université du Québec à Montréal, Montréal, Québec, Canada; b Researcher, Center for Research and Intervention on Suicide, Ethical Issues and End-of-Life Practices, Montreal, Québec, Canada; c Réseau Québécois de Recherche en Soins Palliatifs et de fin de vie (RQSPAL), Quebec, Canada

**Keywords:** Palliative care clinicians, palliative care professionals, burnout, suffering, powerlessness, recognition, interpretative phenomenological analysis

## Abstract

Palliative care (PC) clinicians work alongside people who are at the end of their lives. These patients face death and suffering, which may also cause significant suffering for the PC clinicians themselves. Previous studies suggest that a significant number of PC professionals suffer from compassion fatigue, vicarious trauma and burnout. However, very few studies have attempted to better understand the meaning of PC clinicians’ lived experience of suffering in its complexity and intricacy. Drawing upon Interpretative Phenomenological Analysis (IPA), this study aimed to explore the PC clinicians’ experience of suffering from a phenomenological and existential perspective. In-depth interviews were conducted with twenty-one specialized PC clinicians who were all part of the same multidisciplinary team. Interviews were analysed using IPA. The three emerging essential themes describing the meaning of clinicians’ suffering were 1) Suffering as powerlessness; 2) suffering as non-recognition and 3) easing suffering: the promise of recognition. Result interpretation was based on Paul Ricoeur’s existential phenomenology of suffering and recognition. The conclusion calls for support initiatives and interventions aimed at promoting recognition among PC clinicians on personal, professional, and institutional levels.

## Introduction

The collective attitudes of Western and industrialized societies are often characterized by a denial of death (Aries, 1981; Becker, 1973; Byock, 2002; Crimmins et al., 2020; Tradii & Robert, 2019; Zimmerman, 2004). Contemporary Western values consistently aim to push back the limits of mortality while striving for health, youth, and independence at all costs. By contrast, the ideas of illness, old age, and any related forms of dependence appear senseless and without benefit.

Among other things, these contemporary social attitudes about death and dying seem maintained and supported by the modern medical model (Zimmerman, 2004), which continually prioritizes the prolongation of life through new therapeutic technologies designed to increase longevity, sometimes to the detriment of many quality-of-life (QOL) factors. Consequently, death is often perceived as a failure of medical science or a taboo subject (Byock, 2002). However, the perpetual evolution of curative medicine may only extend life’s temporal limits, meaning that such measures cannot affect the inevitability of death.

The philosophy of palliative care (PC) recognizes death as a natural stage of life and is somehow at odds with the aims of modern medicine (Vachon, 2020). Palliative Care employs a holistic approach based on human values in the accompaniment of dying persons and their relatives (World Health Organization [WHO], 2014). It also differs from traditional biomedical approaches in its adherence to the Whole Person Care model. As such, PC aims to relieve suffering at all levels, including the physical, psychological, social, and existential, while considering the patient’s experience as a whole (WHO, 2014). In this context, Cicely Sanders (founder of PC in the 1960’s) introduced the concept of “total pain”, which refers to the full experiences of dying patients with a focus on “wholeness” and complexity (Ong & Forbes, 2005). In order to fully care for the complex needs of these patients, PC is therefore most often delivered by multidisciplinary teams comprised of members who are exposed daily to such episodes of suffering through the nature of their work (Back et al., 2016). It is now well-known that many of these PC clinicians experience substantial emotional distress after continual exposure to intense suffering, repeated loss, and constant reminders of death (Back et al., 2016; Fillion & Vachon, 2018). Often dubbed “the cost of caring” (Fillion & Vachon, 2018), the emotional and existential issues inherent to the PC practice may actually affect long-term health and well-being for these health care professionals (Back et al., 2016).

Over the past 10 years, an increasing number of researchers have shown interest in better understanding the experiences of PC clinicians (Back et al., 2016). These studies suggest that a significant number also suffer from compassion fatigue (Slocum-Gori et al., 2013), vicarious trauma (Sinclair & Hamill, 2007), and burnout (A. H. Kamal et al., 2019; A. Kamal et al., 2014; Kavallieratos et al., 2017). Certain studies have further suggested that up to 50% of PC clinicians experience distress due to the adversity of working in their field (Kamau et al., 2014). Moreover, the burnout rate seems to be increasing (A. Kamal et al., 2014). While burnout is traditionally conceptualized as a psychological syndrome that arises due to chronic job stressors (Fillion & Vachon, 2018), most recent research in this area has focused on the degree of match/mismatch between the professional environment and the worker’s values or goals (Back et al., 2016; Fillion & Vachon, 2018; Fillion et al., 2017; Harrison et al., 2017). For instance, mismatches are often observed in the PC environment when workloads and insufficient resources interfere with the quality of care professionals desire to provide. Excessive workloads also exhaust PC clinicians and may generate a conflict of values so profound and prolonged that recovery becomes impossible (Fillion & Vachon, 2018).

Some studies have attempted to identify the specific stressors related to the PC work environment, thus aiding efforts to prevent both distress and burnout. For instance, Fillion et al. (2003) described three categories of stressors that affect well-being for PC clinicians. The first contains emotional stressors, which are understood as cumulative grief, exposure to distress expressed by patients and their families, and personal discomfort related to suffering and death (Fillion et al., 2003). The second is comprised of organizational factors such as staff shortages and insufficient resources, which may also cause stress. Lastly, the third relates to professional stressors such as the increasing technologization of care, interprofessional conflicts, and ethical dilemmas (Fillion et al., 2003). Based on these findings, different interventions have been designed to address professional and organizational stressors while also increasing the ability of PC clinicians to cope with these issues. These interventions have taken different forms, the most common of which being support groups, stress management techniques, education/training, meaning-centred interventions, and mindfulness-based stress reduction (Fillion & Vachon, 2018).

Though promising, most interventions designed to facilitate stress coping and resilience among PC clinicians have their shortcomings. First, no intervention has yet more efficiently prevented or healed exhaustion (Hill et al., 2016). Another issue is the lack of an organizational component; that is, no measures have been taken at an institutional level to systematically implement these interventions while ensuring their durability or even availability (Harrison et al., 2017; Vachon & Fillion, 2019). Combined with this, a lack of overall recognition about the need for such support places a disproportionate responsibility on the PC clinicians themselves when attempting to adapt to profoundly challenging environments. Moreover, many interventions are often implemented without any cost-benefit logic, which is fundamentally incompatible with the paradigm of care (Péoc’h, 2016). In this context, some scholars have deemed the lack of clinicians’ support due to budgetary or institutional constraints a form of institutional violence (Péoc’h, 2016).

Recent research has also suggested that PC clinicians may experience suffering through the complex imbrication of factors on many levels, including the institutional, professional, psychological, and existential (Vachon & Fillion, 2019). To the best of our knowledge, very few studies have attempted to better understand this complex experience as a whole in the particular context of institutional violence and death denial. In the spirit of the Whole Person Care approach and PC philosophy, however, there is value and relevance to the conceptualization of suffering in its wholeness and complexity. Such a conceptualization embraces the fundamental and existential character of what it means to experience suffering as a whole person who cares for dying patients.

### Aims

To our knowledge, no studies have attempted to develop an understanding of PC clinicians’ suffering as a lived experience based on the perspectives of the PC clinicians themselves. This study therefore raised the following question: What is the meaning of suffering for PC clinicians? As such, we aimed to derive a better understanding of suffering as a lived, phenomenological experience in order to generate new insights into the elements needed to support and improve both health and well-being for PC clinicians. This study was conducted within a wider collaborative research process that took place in a specialized PC ward at a tertiary medical centre. The main objectives of the project were 1) to better understand the suffering of the PC staff and 2) suggest recommendations for supporting them.

### Theoretical and methodological approaches

This study was conducted through a phenomenological, existential and hemeneutic approach set within a constructivist-interpretative framework (Ponterotto, 2005). The constructivist-interpretative paradigm stipulates that the meaning of an experience stems from a unique co-creation accomplished by the participant and researcher (Ponterotto, 2005). As such, this study investigated both the participants’ lived experiences and the researchers’ subjectivities to better understand the meaning of the experience of suffering for PC clinicians. In this context, a rigorous approach requires the researcher to be both transparent and authentic about their theories and assumptions when taking an active role in exploring and interpreting participant experiences (Morrow, 2005; Tracy, 2010).

In this study, the researchers proceeded with their investigation based on both experiential and theoretical sources of knowledge. First, as a clinical psychologist and researcher specializing in the area of PC, we gained previous clinical and research experience while working alongside dying patients on a specialized PC ward. We (first author) had lived and observed experiences of suffering in the study context. These tacit and experiential understandings of the phenomenon and context were used with proper reflexivity (e.g., journal-keeping and peer-discussions) to ensure that overall comprehension would be enhanced rather than limited (Morrow, 2005; Tracy, 2010).

On a theoretical and methodological level, this study’s phenomenological research stance is anchored in Interpretative Phenomenological Analysis (IPA) (Smith, 2004; Smith et al., 2009; Smith & Osborn, 2003; Tuffour, 2017). Interpretative Phenomenological Analysis is a flexible and versatile approach to exploring, describing, interpreting, and situating the participants’ sense-making of their experiences (Tuffour, 2017). Since it aims to explore and describe in detail participants’ personal lived experience (Smith, 2004), IPA adopts a phenomenological perspective. However, it distinguishes from traditional descriptive phenomenology. IPA stipulates that phenomenological reduction is impossible, and thus rejects the idea of suspending all judgement about an experience (Smith et al., 2009; Tuffour, 2017). In fact, IPA has emerged by identifying more closely with the hermeneutic tradition of phenomenology. Often defined as the science, theory and practice of interpretation (Bleicher, 2017), hermeneutics is also the study of understanding, to decipher meanings (Guzys et al., 2015). Meaning in this context is to be understood as something fluid that is continuously open to new insight, revision, interpretation, and reinterpretation (Smith et al., 2009).

Interpretative Phenomenological Analysis is also often referred to as “double hermeneutics” in that the researcher is making sense of the participants’ sense-making process (Tuffour, 2017). IPA researchers therefore investigate the process of revealing meaning alongside the participant’s way of making sense out of their lived experience. As such, the investigator may take a slightly more active role during interpretation (Smith, 2004; Smith & Osborn, 2003). For reflexive, relational, and ethical reasons, this study paid careful attention to how participants revealed themselves in relation to their experiences of suffering. This was practically accomplished by questioning participants about their interview experiences during the research and integrating those remarks in the final analysis. These complementary steps appeared to be very helpful in identifying the central themes to understand participants experience. Throughout the research process, we followed Tracy’s (2010) eight key markers of quality in qualitative research, as shown and exemplified in Table I.

**Table I.** Tracy’s (2010) eight key markers of quality in qualitative research

Key markers	Means through which criteria were achieved
Worthy topic	Study’s relevance was supported by the palliative care clinical team Supported by the existing literature in the field
Rich rigour	Relevance of the findings in relation to existing theoretical constructs (ex. Recognition, burn-out) Significant sample size for qualitative phenomenological research In-depth interviews by psychologists Discussion with peers
Sincerity	Transparency with regards to the methods (recruitment, interview coding, etc.) Recognition of the researchers’ subjectivity, Keeping a reflexive diary Recognition of study limitations
Credibility	Substantive citations from diverse participants Crystallization (with peers and clinical teams)
Resonance	Evocative representations Transferable findings Result validation with the clinical team
Significant contribution	Conceptually/theoretically Clinical contribution/transferability
Ethics	Procedural ethics (approved by a board of ethics) Situational ethics (interviews conducted by psychologists; referral offered if needed) Relational ethics (included all participants who showed interest; availability of researcher)
Meaningful coherence	Question/paradigm/design and analysis in line with IPA Coherence between literature, data and interpretations

## Methods

### Participants

As mentioned, this study was part of a wider participatory research project conducted with a specific specialized PC team. All members of the specialized PC team with whom the study was conducted were invited to participate in an interview designed to better understand their experiences of suffering. The only inclusion criteria was to be a member of the PC team. Ultimately, 21 of 38 members expressed interest in participating. The final sample included nurses, patient-care attendants, physicians, and members of the psychosocial team. In-depth individual interviews were conducted with each participant. Table II provides the sociodemographic characteristics of the sample.

**Table II.** Sociodemographic characteristics

	Mean (SD)	Range
**Age** **Years of experience in PC** **Means hours/week spent with patients**	43 (13) 9 (8.07) 18.78 (4.22)	21–56 6 months—27 years 16 h—45 h
**Gender** Women Men	**Number participants** *n = 19* *n = 2*	
**Work statut** Part time Full Time	*n = 6* *n = 15*	
**Occupation** Nurse Patient care attendant Member of the multidisciplinary team Physician	*n = 9* *n = 4* *n = 6* *n = 2*	

### Ethical considerations

This study received ethical board approval from the tertiary medical centre where the research took place (Montreal, Canada). The researchers provided study information to all participants both verbally and in writing. Specifically, participants were informed of the study’s purpose, the voluntary nature of their participation, and their ability to withdraw at any time. Further, all participants were assured confidentiality; all analyses were thus conducted with the intention of maintaining integrity for all participating persons. Written informed consent was obtained for each participant.

### Procedure and settings

In-depth, semi-structured interviews were conducted by the main researcher (n = 6) or by a trained psychology PhD candidate (n = 15), under the supervision of the principal investigator. Each interview ranged from 45 minutes to two hours (Mean = 63 minutes). Each participant was interviewed in a private and confidential space of their choosing at their workplace (during or after their shifts) after receiving approval from the PC unit manager. The interview guide was developed by the principal researcher who is also a clinical psychologist specialized in PC. The interview guide was validated by two PC clinicians (not related to the recruited team) and by two qualitative researchers specialized in PC. During the interviews, the participants were invited to share an experience of suffering as a PC clinician. Prompts were used to deepen the exploration: a) what happened? b) how did you feel? c) what was the most difficult? Participants were also questioned about episodes in which their suffering was appeased. Finally, upon concluding, participants were invited to share their experience of the interviews themselves. This was done to ensure situational and relational ethics (Tracy, 2010) and to enrich our understanding of the participants’ meaning-making process. All interviews were audio recorded and fully transcribed. Detailed reflexive notes were taken after each interview. Interviews were also discussed between the interviewer (PhD candidate) and principal investigator (first author).

### Data analysis

IPA methodology (Smith, 2004; Smith & Osborn, 2003) is often described as a form of “guiding light,” this being opposed to a series of rigid steps. The principal investigator (fist author) was responsible for data analysis. She first gained precious insight through lengthy data immersion by reading the transcripts many times and adding detailed reflexive notes. Each transcript was then individually examined to identify meaning units. This step was also accompanied by reflexive note-taking about the ways in which these meaning units were interrelated and/or about any themes that emerged during the analysis. The N’Vivo software was then used to organize the meaning units, which were grouped into overarching themes. This resulted in 12 different themes that described significant dimensions to the experience of suffering.

The researchers then reread all reflexive notes and more closely examined participant interview experiences. Reflexive questions were thus identified, including “For this participant, what was the significance of telling us about their experience of suffering?” Such reflexive questioning is specific to IPA and allowed for a clearer identification of certain themes that were associated with suffering as well as the emergence of three essential themes. The researchers then described the essence of these three themes and supported this description based on participant narratives and specific quotes. All transcripts were then reviewed to ensure that the three emerging themes included all important aspects of the derived participant experiences and that each participant experience was represented.

The current existential and phenomenological literatures were then consulted to enrich and better define the three discovered themes. This review highlighted the writings of French philosopher Paul Ricoeur (1994, 2005)), thus inspiring further investigation to deepen and articulate the overall level of comprehension concerning the three emerging themes. To ensure a collaborative process, these results were shared with all participants and management team members through presentations, a summary report, and blog that was created to record the reactions of all team members (even nonparticipants). This step was taken to ensure credibility and resonance; the ensuing feedback appeared to support existing understandings. Moreover, impressions, reactions, and comments (e.g., “feeling understood,” “feeling moved and touched by the researchers’ words and thoughts,” and “finally, someone understands”) indicated that said comprehension had achieved significant resonance for participants (Tracy, 2010). These findings also supported the study’s relevance and value (at the very least for this team).

## Results

The phenomenological analysis allowed the identification of three essential themes that carried the meaning of suffering for PC clinicians: 1) Suffering as powerlessness; 2) suffering as non-recognition and 3) easing suffering: the promise of recognition. The three themes will be presented and illustrated through participant quotes and reactions to the interview process, which helped clarify the meaning of their experiences.

### Suffering as powerlessness

A vast majority of participants often shared experiences in which they felt powerless when questioned about suffering. Such powerlessness manifested in several different ways and could stem from either specific care practices or general aspects of PC. Indeed, the very nature of PC work entails that clinicians are repeatedly confronted with death and intense suffering, which may cause suffering for the clinicians themselves. However, the particularities of such experiences were found precisely in the feelings of powerlessness generated by the witnessing of episodes in which others were suffering. For example, some participants reported situations in which they felt overwhelmed by their own emotions or, more often, helpless when faced with the suffering of a patient or colleague. One nurse stated the following:

It’s just so difficult to watch someone suffer … it’s not being able to ease this suffering … and to see the family suffering too …^1^ (P3)

Another nurse recalled feeling particularly helpless when faced with the intensity of suffering at a young father’s death:

And I went down to the family room and I saw his wife … When she saw me, she just grabbed me and she wouldn’t stop crying, so I started crying too … I didn’t know what to do … I wish I could do something … find something to say, you know, bring comfort … it was just too much pain … Thinking of the children … It still hurts to think about it … I wish … I don’t know … (P6)

A physician recounted the following about a particularly difficult experiences in which patient suffering could not be alleviated:

In me, I have a kind of little cemetery. All these cases, all these deaths we could have done more for and done better for … It’s like buried deep inside. (P9)

Most PC clinicians also reported experiences of powerlessness and suffering in situations where they did not have the means to provide the care they considered appropriate due to certain constraints and conflicts. For instance, there were some situations in which one or several family members interfered with their loved one’s care by forbidding the administration to use medication that could have relieved the patient’s pain or by refusing to take part in exchanges deemed important and useful for the patient’s care.

Further, for some participants, a specific form of powerlessness stemmed from an imbalance between the level of responsibility PC clinicians felt with respect to patient suffering and their relative abilities to have a positive impact on the patient’s situation. For example, participants witnessed patient suffering while having no authority over the prescription of pain medication or the nature of the related care programme. Circumstances in which PC clinicians were prevented from providing the care they believed was adequate and thereby being unable to relieve suffering often led to feelings of helplessness. One nurse stated the following:

I remember … The patient was in terrible pain, but I couldn’t reach the physician on call. The resident was there but he was on board with what was happening … (P8)

Most PC clinicians shared feelings of complete powerlessness when faced with mandatory organizational and institutional factors that were often incompatible with the holistic-humanistic approach associated with PC. Many PC clinicians confessed that they did not have the means to work according to their values or personal ethical standards of care; some were even prevented from providing care that was congruent with the overall PC philosophy. The inability to provide what was considered “good care” generated powerlessness, which was also expressed as an experience of suffering. PC clinicians recalled several occasions when their care delivery options were limited. Two nurses specifically recounted the following:

To me, I think that the biggest emotional pain comes from not being able to provide the care I would like. Currently, with all the cuts, shortages, imposed changes … On a scale of 0 to 10, I would say I am at 1 out of 10 for what I would like to offer. (P18)

The palliative care culture is already under severe attack from the pressures and demands of the system that we work in … And the focus on technology and efficiency will be even greater … I think that working in such an environment that may not be compatible with what we want palliative care to be and is responsible for a lot of suffering. And it’s all imposed by a large system, even a culture … there’s nothing we can do about it. (P12)

An analysis of these accounts clarified that most PC clinicians often experienced a form of suffering that signified powerlessness when confronted with the sufferings of others. Feelings of powerlessness also emerged when trying to administer care according to the humanistic-holistic values and philosophy of PC.

### Suffering as non-recognition

Most participants also expressed many instances in which their own suffering was ignored, minimized, or denied by themselves, their own relatives, or those in positions of direct power in the professional context. For example, PC clinicians reported the lack of a place or space in which they could be heard and/or deposit their suffering daily:

Everything we have to do, everything we witness, suffering, death … And then, added to that, no support, no one interested in what you’re going through … (P7)

And who do you want me to tell this to? Nobody wants to hear my stories. Everyone finds me harrowing or depressing when I try to confide in them. Even my spouse … Most of the time he can’t even conceive the kinds of situations I’m in … (P1)

Such non-recognition of the suffering inherent to the daily work alongside dying patients in addition to the profoundly existential character of those experiences seemed to plunge PC clinicians into an isolation that likely deepened their suffering. In certain cases, this type of non-recognition was manifested as a form of closeness or even a disconnect with their own experiences. One participant related the following:

Every time I lose a patient, I think of my children … I come back at night, I play with them … I try to enjoy the moment I have with them, to feel all of my love … And in the background, there’s always this anxiety … It can happen to anyone. What if it happened to me? I can’t think about that, I become too anguished. I try to continue, to not think about it, to not talk about it. I bury it and I go on. (P11)

In other cases, it was precisely the impossibility to relate and share their experience that caused suffering to PC clinicians, thus bringing about a sort of emotional repression and denial of the existential nature of accompanying patients during end-of-life care and of the inherent suffering thus caused. Another participant stated the following:

It seems necessary for us to have real psychological support … Real moments to talk, time to sit down, name our fears, our frustrations … To talk about what is happening, about patients who are dying, what it’s doing to us, what it means to us … We’re human … We see all kinds of things that echo with our story, our personal lives. We never have a moment to talk to each other, tell our stories, cry, hug. We pile it on, we try to integrate it, to digest it … But we end up hiding it and forgetting our emotions. (P14)

It largely seemed that non-recognition of the specific PC approach at an institutional level correlated to a cultural denial of the finite character of life that echoed the sufferings and isolation of dedicated PC clinicians.

### Suffering and the promise of recognition

Though experiences of non-recognition were revealed as essential elements to PC clinician suffering, we were also touched by participant accounts in which their suffering was appeased. Almost unanimously, PC clinicians reported that instances in which patients and families expressed recognition were profound sources of well-being, satisfaction, and energy:

I get involved and those situations for me are like a gift, you know … In those moments, I not only feel valued as a person, but confirmed and deeply nourished as a spiritual being. (P17)

… My goodness … I do have moments like that. When I do something for the family and they’re really grateful. That’s deeply nourishing. (P2)

Patients and families, they too sometimes will say: “Oh, you’re working today, how nice!” You know? Like they acknowledge the fact that I’m there and they appreciate me … (P16)

Yes, I remember the words of a family … They told me: “We will never forget the kindness and the gentleness of the care you provided my mother with.” You know, this helps, having the feeling you have done something good … (P21)

The sense of recognition and value may also work as a source of well-being when such comments come from peers or colleagues:

Sometimes I’m told by a colleague: “Oh, I’m so happy you’re here!” You know? It encourages me. I always give 100% of myself, but when I hear that, I want to give 200%! I want to double that! I can’t help but feel good inside. (P13)

Experiences of such recognition may also be revealed through the possibility to confide in someone else; that is, relating one’s own experiences to a receptive colleague:

That night I was able to talk with a colleague that was there [at work] … I told her how much yesterday was a difficult day … and she listened … and she only said: “Wow, I can imagine how tough this must have been … ” Just that … just that … made me feel good. (P16)

All participants mentioned the need to talk about their subjective experiences while being heard and recognized in their suffering. There were many verbal and non-verbal manifestations of profound gratitude towards the researchers, first for taking interest in PC clinician experiences, and second for offering a space in which they could recount their experiences while being seen and recognized as both clinicians and individuals who felt suffering. Further, this gratitude gave rise to a hope for the renewal and cultivation of these spaces of recognition. Some participants also wished for their words to be heard wherever they could find power over suffering while receiving legitimacy and recognition from their institution.

Participants revealed that powerlessness and non-recognition comprised the essence of the suffering they experienced and expressed as PC clinicians. Indeed, their accounts during the interviews and those about the interview process itself equally brought these themes to light. This research relied on two essential works by Paul Ricoeur to deepen current essential understanding of PC clinician experiences while enriching both the phenomenological and existential outlooks related to suffering and recognition.

## Discussion

A significant portion of Paul Ricoeur’s work is dedicated to the experience of suffering. Ricoeur clearly distinguished pain from suffering, defining suffering as a concept involving reflexivity, language, or the relationship with oneself. Ricoeur asserted that suffering was defined by how one recognized themselves and others (Ricoeur, 1994). He also argued that suffering was linked to the awareness of self-limitation vis-à-vis oneself and others. Ricoeur thus suggested that the core (the heart) of suffering was intertwined with limitation and, therefore, with a sort of helplessness or powerlessness (Ricoeur, 1994). He continued in his exploration of suffering in terms of powerlessness by describing suffering impairments in regard to the powers to say, do, tell, and consider oneself as a moral agent (Ricoeur, 1994).

This conceptualization provides a better understanding of how PC clinicians experience suffering. The impossibilities of a) relieving the suffering of patients and their loved ones, b) healing according to their values due to institutional constraints, and c) making their suffering heard as a PC clinician were all examples of limitations. These factors impair the PC clinician in his or her capacity to do, say, or act at the height of his or her own moral convictions. In this study, PC clinicians reported the impossibility of telling and sharing their experience at the bedside of dying patients while in the workspace or even in the context of intimate relationships under the pretence that such a reality was too distressing for others. These issues significantly contributed to their suffering. Indeed, the impossibility of sharing such experiences not only contributed to denial and making the problem both invisible and indescribable, but also caused a sort of negation of the PC clinician’s identity (i.e., that of the PC clinician, who bravely chooses to confront daily instances of death and suffering while trying to make sense of it all alone) (Vachon et al., 2012). Identity negation then results in the impossibility of telling one’s stories of confronting death and suffering, which also likely affects the PC clinician’s ability to recognize and value themselves.

The powerlessness to say, do, tell, value, and recognize oneself can also be understood in the context of fundamental human powerlessness in the face of death. On one hand, PC clinicians are forced into silence because their stories are likely to awaken death anxiety of others (Yalom, 2008). The impossibility of telling and sharing experiences likely isolates the PC clinician from his or her own existential anguish and thus limits his or her ability to think about and recognize their own experiences of suffering.

On the other hand, recognition of fundamental powerlessness in the face of death is at even greater odds with the hegemonic vision of modern medical omnipotence, in which PC clinicians operate daily (Vachon, 2020). Indeed, although the PC unit occupied a physical space at a highly specialized university hospital centre, the holistic and humanistic philosophy aimed at recognizing the inevitability of death suffered from a serious lack of recognition within the care institution at that time (Vachon, 2020). In its focus on technological advances and the avowed purpose of prolonging life, the vindictive culture of modern medicine relegates death to failure and taboo. The end of life is thus deprived of meaning and value in the eyes of the highly specialized medical institution (Byock, 2002). It is thus necessary to question PC clinicians who operate within the taboo and marginalized PC space.

In concrete terms, the PC clinician stories of powerlessness found in this study showed significant suffering. This may be a form of helplessness in the face of institutional rules that are often inconsistent with the PC philosophy that inhibits the ability of clinicians to provide good care. In addition, the lack of a communication channel between PC clinicians and authorities in which there is power to influence care conditions can also be understood as a reflection of the lack of recognition about PC clinicians, their experiences, and the value of their devotion to end-of-life patients. A form of rupture (Bourgeois-Guérin, 2012) may thus form between the PC clinician and the institution, thereby relegating the PC clinician to forms of anonymity and silence that further isolate them in their suffering; this may ultimately lead to a form of rupture that is all the more painful for PC clinicians in relation to themselves (Bourgeois-Guérin, 2012).

If PC clinician suffering is understood as a fundamental experience of impotence (Ricoeur, 1994) and rupture (Bourgeois-Guérin, 2012), this analysis further suggests it is also rooted in ruptures of recognition (i.e., those of the inescapable character of our finite existence, of institutional acknowledgement, of the self as a suffering being, and of the existential character of the PC clinician’s experience). In return, listening sessions among colleagues, the gratitude expressed by families and the possibility of recounting suffering within the research space may allow recognition to circulate. PC clinicians should be heard, attempted to be understood, and considered, thus establishing a relationship of sympathy and exchange that can build and renew the feeling of recognition (Métraux, 2007).

In this respect, Ricoeur’s (2005) thoughts are equally rich in their ability to clarify the current understanding of how PC clinicians experience suffering. In his book “The course of recognition” (Ricoeur, 2005), Ricoeur attempted to list the multiple meanings of the term; three were explicitly defined, including 1) recognition as identification (i.e., the ability to identify previously known things or people in one’s environment), 2) recognition of oneself (i.e., the particular recognition of one’s capacities and powers to say, do, act, and tell about one’s responsibilities), and 3) mutual recognition. Each way of understanding recognition corresponds to a way of denying it, which constitute forms of contempt for and an exacerbation of suffering (Métraux, 2007).

Above all, the recognition of oneself in the capacity to say and act is infringed upon during the sufferings and helplessness of PC clinicians. Such attacks are likely to cause breaks in the PC clinician’s relationship with himself and his or her social circle. For Métraux (2007), it is especially through mutual recognition that one can reform the link to the self. Mutual recognition must, however, be accomplished through exchanges of either actions or words. Métraux (2000) spoke of these exchanged words as gifts of precious words. These are words that both attest and testify to incompetence, helplessness, and suffering. Talking about powerlessness and non-recognition are already parts of exchanges that are likely to aid in the circulation of recognition. Words that recognize institutional violence (Péoc’h, 2016) or the social and cultural denials of death (Byock, 2002; Crimmins et al., 2020; Tradii & Robert, 2019; Vachon, 2020) that condemn PC clinicians to isolation are also gifts of recognition. Recognition does not end there, however. The issue of mutual recognition also includes gratitude. Moreover, this study found that it was precisely the words of gratitude from patients to their PC clinicians that enabled recognition to circulate within the caregiving environment. Gratitude invites us to raise the question of recognition by expressing precious words to PC clinicians in order to further express the beauty of their dedication, the courage of their vocation, and the value of their care. This type of gratitude should circulate within professional teams to help them overcome the current lack of social, cultural, and institutional recognition.

### Clinical implications

Current phenomenological and existential understandings of the suffering experienced by PC clinicians invites researchers to rethink both the experiences and available support. For example, professional burn-out and compassion fatigue syndromes afflict a disquieting proportion of PC clinicians (A. H. Kamal et al., 2019; A. Kamal et al., 2014; Kavallieratos et al., 2017). These are already cited in the scientific literature and should be viewed from the perspective of breaks in the recognition of the self and others. As such, Métraux (2007) also used the terms “recognition diseases” to make sense of certain clinical configurations that can be questioned in their complex meanings. This is especially true when one is interested in individuals who are operating in marginalized areas, such as PC.

To date, the scientific literature offers a range of support interventions designed for PC clinicians. These essentially focus on teaching tools that can develop resilience to workplace stresses (Back et al., 2016; Fillion & Vachon, 2018; Hill et al., 2016; Vachon & Fillion, 2019). However, these interventions are likely to negate the suffering that is inherent to the act of supporting end-of-life patients by stressing the PC clinician’s responsibility to adapt to his or her own environment. They also negate the institutional responsibility to recognize the experiences of PC clinicians working at their facilities. The understanding this study seeks to promote encourages a focus on personal, professional, and institutional support interventions designed to promote recognition. Not only would this be necessary to ensure that words of suffering and gratitude circulate within these institutions, but it would also to encourage genuine expressions of gratitude for the courage and devotion of the PC clinicians. For the institution, this would be a matter of recognizing and explicitly valuing the related needs of PC clinicians while supporting personal and professional initiatives that allow recognition to circulate.

### Methodological considerations

The rupture of recognition as the essence of the suffering experience of PC clinicians has been revealed through this research project. Indeed, participating PC clinicians expressed a great deal of gratitude towards this study’s research team for identifying, naming, caring about, and listening to their expressions of suffering. Rooted in collaborative research, the researchers also shared their findings with these PC clinicians. The emotional resonance they showed in being touched by such an understanding of their issues further provided this research with both rigour and credibility (Tracy, 2010). The density of collected stories, reflexive proximity of the researcher to the health care setting, and authenticity with which all derived stories are presented here also extend value to the caregiving experience (Tracy, 2010). The result is a phenomenological and existential understanding of PC clinician suffering that is enriched by Ricoeur, thus allowing a direct expansion of the existing literature. Moreover, the privileged posture of the principal investigator, also having clinical experience as a PC clinician (psychologist), may have contributed to generate insights about the meaning of suffering while caring for dying individuals. As Ricoeur linked phenomenology and hermeneutics by explaining that experience and meaning are closely intertwined, the revealed meaning of suffering among PC clinicians may also relate to the researcher’s experience of suffering. In this research, the dynamism of interpretation and personal reflection resounds excellently with the hermeneutic circle model that deals with the dynamic relationship between the “part” and the “whole” (Tuffour, 2017). In relation to IPA, the “part” corresponds to the encounter with the participant in a research project, and the “whole” the drawing of knowledge and experience of the researcher (Smith et al. 2019; Tuffour, 2017).

Despite the significant contributions of this study, there were also some limitations (Tracy, 2010). First, the research was conducted in a single PC unit when the team was experiencing major organizational changes. While the understanding thereby developed of the PC clinician experience is likely transferable to other caregiving situations experienced by PC clinicians, it remains part of the specific context in which it emerged. Nevertheless, this study’s approach reflects a concern for both rigour and notable ethics, as evidenced by the various measures implemented to ensure that it adhered to the rigorous criteria established by Tracy (2010). Further, the collaborative aspect and stated concern for all participants combined with the theoretical, experiential, and procedural transparency of all processes contributed to the value of its conclusions.

### Conclusion

PC clinicians must work alongside people who are at the end of their lives. These patients face death and suffering, which may also cause significant suffering for the PC clinicians themselves. This study’s phenomenological and existential analyses of the stories related by suffering clinicians working in PC revealed that both powerlessness and non-recognition were at the core of these experiences. Such suffering was notably manifested through instances in which it was impossible to relieve patient suffering, to open up about experiences of one’s own suffering, and to recognize oneself as a suffering person. Experiences of suffering and helplessness in this regard can also be understood within the larger institutional and cultural contexts, in which death is taboo and marginalized. However, enhanced perspectives can be obtained regarding the need for PC clinician support by understanding their suffering and recognizing how workplace deficits may cause distress. As such, institutional spaces should be provided to facilitate and promote mutual recognition and gratitude, which can thus circulate in a way that nourishes and heals the clinician in the context of their brave daily confrontations with the finite character of life and the suffering expressed by others.

## Note

1. Occasionally, participant statements are slightly modified to facilitate reading. For instance, we did not included verbal tics or stuttering.
